# Recent advances in the study of anesthesia-and analgesia-related mechanisms of S-ketamine

**DOI:** 10.3389/fphar.2023.1228895

**Published:** 2023-09-14

**Authors:** Jian-shun Zhou, Guan-fa Peng, Wei-dong Liang, Zhen Chen, Ying-ying Liu, Bing-yu Wang, Ming-ling Guo, Yun-ling Deng, Jun-ming Ye, Mao-lin Zhong, Li-feng Wang

**Affiliations:** ^1^ The First Clinical Medical College of Gannan Medical University, Ganzhou, China; ^2^ Department of Anesthesiology, First Affiliated Hospital of Gannan Medical University, Ganzhou, China; ^3^ Ganzhou Key Laboratory of Anesthesiology, Ganzhou, China

**Keywords:** S-ketamine, NMDA, ketamine, anesthesia, analgesia, AMPA, opioid receptor

## Abstract

Ketamine is a racemic mixture of equal amounts of R-ketamine and S-ketamine and is well known to anesthesiologists for its unique dissociative anesthetic properties. The pharmacological properties of ketamine, namely, its sympathetic excitation, mild respiratory depression, and potent analgesia, are still highly valued in its use as an anesthetic for some patients. In particular, since its advent, S-ketamine has been widely used as an anesthetic in many countries due to its increased affinity for NMDA receptors and its enhanced anesthetic and analgesic effects. However, the anesthetic and analgesic mechanisms of S-ketamine are not fully understood. In addition to antagonizing NMDA receptors, a variety of other receptors or channels may be involved, but there are no relevant mechanistic summaries in the literature. Therefore, the purpose of this paper is to review the mechanisms of action of S-ketamine on relevant receptors and systems in the body that result in its pharmacological properties, such as anesthesia and analgesia, with the aim of providing a reference for its clinical applications and research.

## 1 Introduction

Ketamine is an N-methyl-D-aspartate (NMDA) receptor antagonist consisting of a racemic mixture of equal amounts of R-ketamine and S-ketamine. The affinity of S-ketamine to NMDA receptors is three to four times higher than that of ketamine in general, with stronger analgesic effects and fewer psychotomimetic effects (Zanos et al., 2018). As an anesthetic, S-ketamine alone is approximately two times more effective than ketamine and approximately triple that of R-ketamine alone (Zanos et al., 2018). S-Ketamine acts rapidly. The peak concentration value is reached approximately 67 s after 0.5 mg kg^−1^ intravenous injection, with a half-life of 287.50 ± 110.20 min (Wang et al., 2019). Analgesic effects can be produced at low doses, and both anesthetic and potent analgesic effects can be produced at high doses (Peltoniemi et al., 2016). In clinical applications, S-ketamine has the following advantages: 1. Intramuscular injection can take effect rapidly and is indicated for uncooperative patients such as neonates and children (Scheppke et al., 2014; Gao et al., 2016; Zanos et al., 2018); 2. S-ketamine is a rational option for the induction of anesthesia in critically ill patients with shock or hypotension because it excites the sympathetic nerves (Jabre et al., 2009; Morris et al., 2009); 3. S-ketamine can relax the bronchi, which is advantageous for the induction of anesthesia in asthmatics (Robinson, 1985; Garner et al., 2022); 4. The strong analgesic efficiency and mild respiratory depression of S-ketamine provide a significant increase in safety when used for postoperative analgesia (Jonkman et al., 2018; Rugg et al., 2021). S-ketamine clearly has unique advantages for clinical applications in specific patients; however, some side effects associated with the use of S-ketamine may occur, such as psychotomimetic symptoms, nausea and vomiting, impaired vision, and dizziness (Zanos et al., 2018). S-ketamine produces a multitude of pharmacological effects that are closely related to its complex mechanism of action. In addition to antagonizing NMDA receptors, it also interacts with opioid receptors, the monoamine system, the cholinergic system and AMPA receptors. Understanding the pharmacological effects produced by interactions of ketamine with various receptors and systems can provide a theoretical basis for the rational use of S-ketamine.

## 2 NMDA receptors

### 2.1 Structural basis of NMDA receptors

NMDA receptors are ionotropic glutamate receptors that are usually composed of three fundamental subunits, GluN1, GluN2, and GluN3, which are widely found in the central nervous system (Chou et al., 2020). NMDA receptors plays a crucial role in synaptic transmission and synaptic plasticity and may be involved in learning and memory (Chou et al., 2020). In anesthesia, NMDA receptors also play an important role in sedation, analgesia, and amnesia (Petrenko et al., 2014). The anesthetic and analgesic effects of S-ketamine, as an NMDA receptor antagonist, are closely related to these functions of NMDA receptors.

**FIGURE 1:**
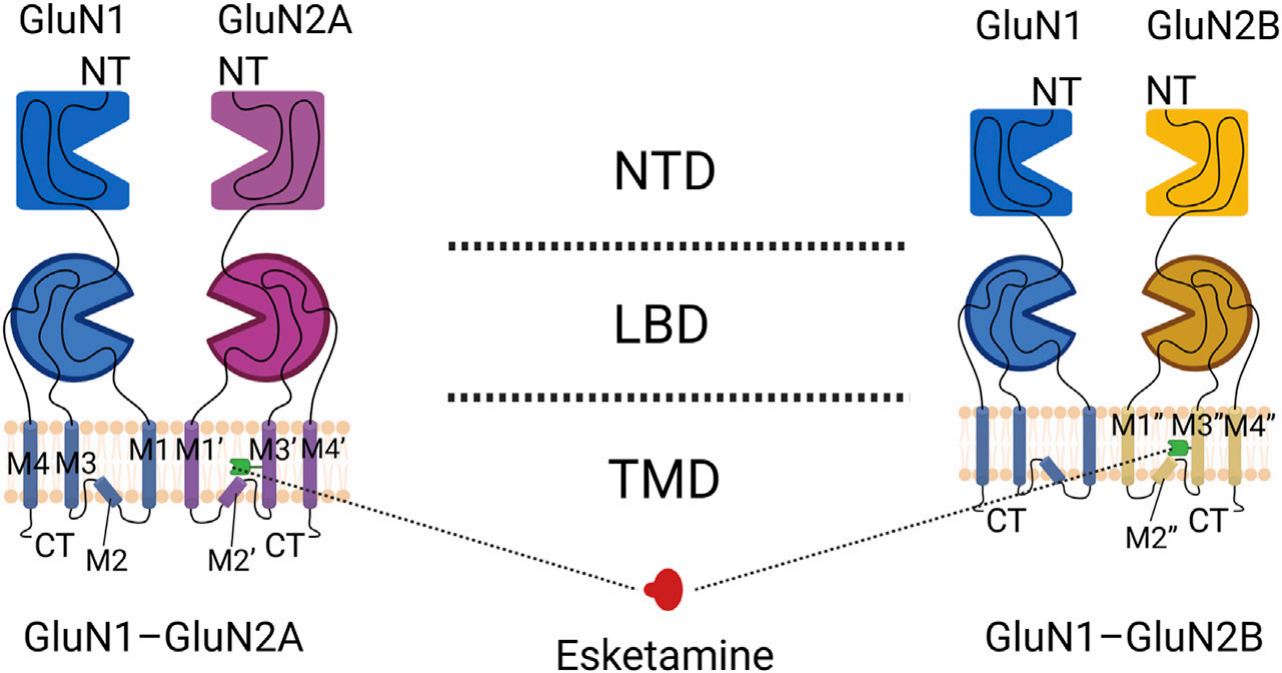
Domain organization of NMDA receptor subunits. The GluN1-GluN2A heterodimer is shown on the left, and the GluN1-GluN2B heterodimer is shown on the right. Blue indicates the GluN1 subunit, purple indicates the GluN2A subunit, yellow indicates the GluN2B subunit, and the green receptor indicated by the dotted line is the binding site of S-ketamine.

**FIGURE 2:**
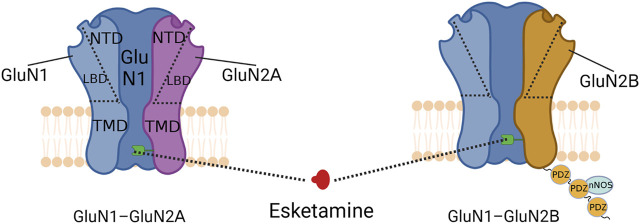
Schematic diagram of NMDA receptor structure. The green receptor indicated by the dotted line is the binding site of S-ketamine. The three PDZ structural domains linked below the GluN2B subunit form PSD-95, which can interact with nNOS to regulate the production of NO.

**FIGURE 3:**
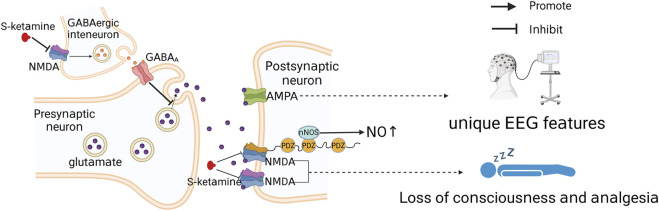
Simple diagram of S-ketamine producing anesthesia and analgesia. S-ketamine acts on GluN1-GluN2A and GluN1-GluN2B subunits and blocks NMDA receptor channels, which is the basis of S-ketamine-induced loss of consciousness and analgesia. Moreover, inhibition of presynaptic NMDA receptor channels can lead to deinhibition of glutamatergic neuronal activity and indirect activation of AMPA receptors.

The GluN3 subunit is strongly associated with central nervous system disorders, but its relationship to S-ketamine remains unknown (Pérez-Otaño et al., 2016). A functional NMDA receptor is usually a tetrameric ion channel consisting of 2 glycine-binding essential subunits, GluN1, and 2 glutamate-binding regulatory subunits, GluN2 (with subunits A–D) (Hansen et al., 2021). S-ketamine mainly acts on GluN1-GluN2A and GluN1-GluN2B subtypes in the adult brain, and its typical structures include the extracellular amino terminal domain (ATD) and the ligand-binding domain (LBD), as well as the transmembrane domain (TMD) of ion channels embedded in the lipid bilayer (Karakas and Furukawa, 2014; Zhang et al., 2021). The LBD is usually a binding site for glycine or glutamate and also binds to some metabotropic modulators (L-689,560,SDZ-220-040); the ATD binds mainly to a subset of metameric regulators (polyamines, phenylethanolamines, or Zn^2+^) that regulate ion channel TMD opening and closing (Karakas et al., 2009; Chou et al., 2020). The TMD consists of four hydrophobic segments (M1 to M4) (Figures 1, 2 **)**, three of which are α-helical transmembrane structural domains (M1, M3 and M4), with the fourth being the short foldback loop (M2) that faces the cytoplasm and does not span the membrane (Zhang et al., 2021). S-ketamine interacts with specific amino acids in the TMD (leucine 642 on GluN2A, leucine 643 on GluN2B, and asparagine 616 on GluN1) when NMDA receptor channels open and forms hydrophobic and hydrogen bonds (Zhang et al., 2021), which in turn alter ion channel structure, inhibit Ca^2+^ hyperpermeability, decrease channel opening time as well as the frequency, and prevent neuronal activation required for a state of consciousness, thus producing an anesthetic effect (Lisek et al., 2020). Research has shown that the dissociative anesthesia induced by ketamine may also mediate some of its analgesic properties (Gitlin et al., 2020; Olofsen et al., 2022). In conclusion, as an NMDA receptor antagonist, the blockade of NMDA receptor channels is the basis for producing anesthesia and analgesia, with NMDA receptor channels interacting with other receptors or channels to exert various pharmacological properties (Figure 3).

### 2.2 GluN2A subunit and hypnosis

GluN2 includes four subunits, GluN2A, GluN2B, GluN2C, and GluN2D, which largely determine physiological functions such as NMDA receptor agonist affinity, single-channel conductance, Ca^2+^ permeability, and Mg^2+^ sensitivity (Bai et al., 2013). S-ketamine binds to leucine on the GluN2 subunit and plays an important role in antagonizing the NMDA receptor (Zhang et al., 2021). GluN2A and GluN2B subunits are widely present in the adult mammalian cerebral cortex and hippocampus and are the most common subunits (Bai et al., 2013). In contrast, GluN2C and GluN2D subunits are more often found in the midbrain and hindbrain and have low receptor opening rates and single channel conductance; due to these properties, current studies are mainly focused on GluN2A and GluN2B subunits (Clarke and Johnson, 2006; Bai et al., 2013). The GluN2A subunit is essential for synaptic plasticity and memory in mammals (Yang et al., 2022), and mutant mice with deletion of this subunit gene are resistant to the hypnotic effects produced by ketamine (Petrenko et al., 2004; Sato et al., 2004). This may be caused by the deletion of the GluN2A subunit that disrupts the function of NMDA receptors, which in turn attenuates the ketamine-induced hypnotic process (Petrenko et al., 2014). However, it has been suggested that this resistance may also be a result of other neurotransmitter alterations arising from the deletion of the GluN2A subunit (Petrenko et al., 2014). In a study of inhalation anesthetics, deletion of the GluN2A subunit was found to increase the release of dopamine as well as 5-hydroxytryptamine in the frontal cortex and striatum of mice (Miyamoto et al., 2001), suggesting that this secondary increase in monoaminergic tone is the main reason for the resistance of GluN2A deletion mice to the hypnotic effects of these inhaled anesthetics (Petrenko et al., 2010; Petrenko et al., 2013). However, the use of ketamine itself induces an increase in monoaminergic tone (Hashimoto et al., 2017; Pham and Gardier, 2019), which does not affect the anesthetic effect produced by ketamine. Therefore, the resistance of GluN2A deletion mice to ketamine-induced hypnosis is more likely the result of disruption of NMDA receptor function following deletion of the GluN2A subunit.

### 2.3 GluN2B subunits and analgesia

 While the GluN2A subunit of NMDA receptors is mainly present within synapses and mediates learning, memory and synaptic plasticity, GluN2B is mainly present in extrasynaptic NMDA receptors and mainly mediates glutamate excitotoxicity, which can cause deleterious effects on neuronal function (Sun et al., 2017; Tian et al., 2021). The GluN2A subunit may not be involved in mediating the ketamine analgesic process, as it does not produce significantly different analgesic effects in wild-type and GluN2A subunit-deficient mice (Petrenko et al., 2006). In contrast, the GluN2B subunit plays a key role in central sensitization and in various types of chronic pain, both neuropathic and inflammatory (Wei et al., 2001; Wang et al., 2021). Transgenic mice that overexpress the GluN2B subunit exhibit selectively enhanced persistent and abnormal pain (Wei et al., 2001), whereas knockdown of the GluN2B subunit prevents the development of abnormal pain (Wang et al., 2021). This relationship of the GluN2B subunit to chronic pain may be the essential reason for the promising results of ketamine in the treatment of clinical chronic pain (Marchetti et al., 2015).

Chronic pain regulation by GluN2B subunits may be associated with the scaffolding protein postsynaptic density-95 (PSD-95) (Figure 3). PSD-95 is a membrane-associated protein consisting of three PDZ structural domains (Kim and Sheng, 2004) that interacts with nitric oxide synthase (nNOS) to modulate NO, a pain transmitter (Zhou et al., 2010; Sarmah et al., 2022). Since the N-terminal PDZ structural domain of PSD-95 and the C-terminus of the GluN2B subunit are interactable, the GluN2B subunit may indirectly regulate NO production through PSD-95 and thus the development of chronic pain (Li et al., 2022). This possibility has been further validated by the fact that chronic pain can be reduced by disrupting the interaction between the GluN2B subunit and PSD-95 (D'Mello et al., 2011; Li et al., 2022). Therefore, S-ketamine likely inhibits NO release via the GluN2B/PSD-95/nNOS pathway to reduce pain signaling and prevent the onset of chronic pain. In addition, pain inputs to the dorsal horn of the spinal cord stimulate the release of the neurotransmitter glutamate and activate NMDA receptors, which underlie the transition from acute to chronic pain (Inquimbert et al., 2018). Antagonism of NMDA receptors might inhibit this transition process.

Current studies on the relationship between ketamine and the GluN2 subunit are mainly focused on the observation of changes in potency in the knockout mouse model after drug administration that can indirectly reflect whether ketamine acts through this subunit. However, some limitations exist in all of these studies because the knockout mouse model may have secondary alterations in other neurotransmitter systems; such alterations can have direct or indirect interactions with the drug and affect the results of the experiment. Moreover, NMDA inhibition of different subunit compositions is not isolated; even selective inhibition of extrasynaptic GluN2B subunit-containing NMDA receptors may still antagonize synaptic GluN2A subunit-containing NMDA receptors (Tymianski, 2011). As a result, mice with a knockout mutation for only one subunit likely also have altered expression of the other subunits, and the antagonistic effect of S-ketamine on both subunits adds to the uncertainty of the resulting findings.

## 3 AMPA receptors

### 3.1 The role of AMPA receptors

Ionic glutamate receptors include NMDA receptors, α-amino-3-hydroxy-5-methyl-4-isoxazole propionic acid (AMPA) receptors and kainate receptors (Chou et al., 2020); AMPA regulates the opening of NMDA receptor channels (Hansen et al., 2021). NMDA receptors are dual-gated channels regulated by both membrane potential and other neurotransmitters (Hansen et al., 2021). At a resting membrane potential, physiological levels of Mg^2+^ can block NMDA receptor ion channels (Mayer et al., 1984), and the effect of Mg^2+^ blockage can only be eliminated when glutamate released from the presynaptic membrane acts on AMPA receptors, enhancing ion flow through AMPA receptor channels and causing local depolarization of the postsynaptic membrane adjacent to NMDA receptors (Nowak et al., 1984; Hansen et al., 2021). NMDA interacts with AMPA receptors, and antagonism of NMDA by S-ketamine may also activate AMPA receptors. However, studies on the molecular structure of ketamine and AMPA have shown that amino acid differences in the key sites of AMPA receptors may lead to disruption of the ketamine spatial structure and weaken the space-sensitive hydrophobic contacts, which may result in the selective binding of S-ketamine to NMDA but not to AMPA receptors (Zhang et al., 2021). However, it has also been found that NMDA receptor blockade reduces GABA release from gamma amino-butyric acid (GABA)-ergic interneurons, leading to deinhibition of glutamatergic neuronal activity (Gerhard et al., 2020) and thus indirect activation of AMPA receptors. In addition, the S-ketamine metabolite (2R, 6R)-hydroxynorketamine can also exert sustained activation of AMPA receptors (Zanos et al., 2016). The signaling enhancement after AMPA receptor activation can cause an increase in thalamic and cortical synaptic transmission (Bieber et al., 2022), which may partially explain the different electroencephalographic (EEG) characteristics of the anesthetized state induced by ketamine *versus* those induced by other anesthetics (Purdon et al., 2015). The dissociative anesthesia produced by ketamine is an abnormally altered state of consciousness (Corssen and Domino, 1966; Bieber et al., 2022), and the increased thalamic and cortical synaptic transmission induced by ketamine may also contribute to its production of dissociative anesthesia (Bieber et al., 2022).

### 3.2 The effect of Ca^2+^ on AMPA

The effects of S-ketamine may be indirectly modulated by Ca^2+^. Modulation of Ca^2+^ by S-ketamine binding to the GluN2B subunit in the ionotropic glutamate receptor NMDA may lead not only to sustained activation of AMPA receptors (Kavalali and Monteggia, 2012; Zhang et al., 2021) but also to activation of glutamate-independent pathways that induce Ca^2+^ inflow as well as calcium pool mobilization, resulting in elevated Ca^2+^ concentrations in the cytoplasm of neurons in the brain (Lidow, 2003). Elevated Ca^2+^ concentrations can in turn modulate dopamine release and increase D2 receptor activity, causing psychiatric symptoms (Lidow, 2003; Lisek et al., 2016). In addition, Ca^2+^ modulation may also be involved in S-ketamine-induced euphoria and sedation, which were found to be inhibited by the L-type calcium channel antagonist nimodipine in a clinical trial (Krupitsky et al., 2001).

## 4 S-ketamine and opioid receptors

### 4.1 Involvement in analgesic effect

Opioids have a pronounced analgesic effect and are widely used in clinical practice as classic analgesic drugs (Stein, 2016). The classical opioid receptors include μ, δ, and κ receptors, and as early as 1999, Hirota et al. discovered the interaction of ketamine with these three types of opioid receptors, with S-ketamine being two to three times more potent than R-ketamine at μ and κ receptors; this potency at the μ and κ receptors may be one of the factors contributing to the stronger analgesic potency of S-ketamine than R-ketamine (Hirota et al., 1999). One study investigated the antagonistic effect of ketamine on analgesia by administering nonselective and selective μ, δ, and κ receptor antagonists to mice. Interestingly, κ receptors exhibit negative results in both central and peripheral analgesic antagonism tests in mice, which suggests that the analgesic effects of ketamine are mainly produced through μ and δ opioid receptors, while κ receptors are not involved (Pacheco Dda et al., 2014; Petrocchi et al., 2019). During the study, researchers found that ketamine induced an increase in endogenous opioids to synergize analgesia (Pacheco Dda et al., 2014; Petrocchi et al., 2019). This increase in endogenous opioids (e.g., beta-endorphin) may originate from the stimulation of the pituitary cell line by ketamine, and this stimulation can also lead to a delayed secretion of beta-endorphin, resulting in a longer duration of analgesia than it is expected to be pharmacokinetic with ketamine (YaDeau et al., 2003).

### 4.2 Other possible mechanisms affecting opioid receptors

**FIGURE 4:**
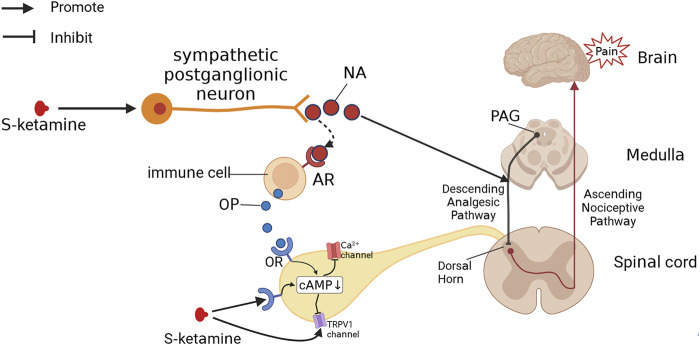
S-Ketamine produces analgesia by other pathways. S-ketamine excites sympathetic nerves, leading to the release of NA. NA both activates the descending analgesic pathway, which produces analgesic effects, and binds to adrenoceptors (ARs), promoting the release of opioids (OPs) from immune cells. Both S-ketamine and OP can bind to the opioid receptor (OR), decreasing intracellular cAMP levels and thus blocking Ca^2+^ channels and TRPV1 channels to produce an analgesic effect.

The effect of S-ketamine on opioid receptors may also be related to the effects of opioid receptor activation (Figure 4). Opioid receptor activation reduces intracellular cAMP production and decreases Ca^2+^ channel and TRPV-1 channel activity in the spinal dorsal root ganglion (DRG), which attenuates pain signaling and produces an analgesic effect (Stein, 2016; Reeves et al., 2022). Long-term opioid use can lead to intracellular “cAMP rescue”, which reduces cAMP concentrations to normal levels, thereby reducing the analgesic effects of opioids and causing drug tolerance (Kibaly et al., 2017; Mizobuchi et al., 2022). According to the study, ketamine activates G-protein-coupled receptor kinase 2/3, activating the μ receptor phosphorylation site, thereby reducing the intracellular cAMP concentration and inhibiting the onset of “cAMP rescue”, which may be one of the reasons why ketamine can ameliorate tolerance to long-term opioid use (Kibaly et al., 2017; Mizobuchi et al., 2022). Notably, ketamine also enhances β-arrestin recruitment during this process and increases the amount of β-blocker binding to the tail conformation of the μ-receptor (which induces resensitization), which improved desensitization (Cahill et al., 2017; Mizobuchi et al., 2022). In addition, opioid receptor activation also inhibits excitatory postsynaptic currents evoked by glutamate receptors in the spinal cord (Stein, 2016), so antagonism of the ionotropic glutamate receptor NMDA by S-ketamine may indirectly produce a synergistic effect of opioid receptor activation and exert analgesic effects. In contrast, S-ketamine can also interact with specific amino acids of the GluN1 and GluN2B subunits to form hydrophobic and hydrogen bonds (Zhang et al., 2021), which in turn form complexes with TRPV-1 receptors in the DRG via these two subunits to enhance TRPV1 receptor signaling, causing the opposite effect to opioid receptor activation and leading to enhanced mechanical pain (Lee et al., 2012a; da Costa et al., 2020).

### 4.3 Other effects of opioid receptors

S-ketamine has some advantages over opioids, such as mild respiratory depression and lack of induced nociceptive hypersensitivity. Although it has been suggested that the interaction of S-ketamine with μ receptors may lead to respiratory depression, low doses of S-ketamine in clinical practice do not provide significant respiratory depression, with respiratory depression only occurring with high or rapid doses (Sarton et al., 2001; Zanos et al., 2018). In addition, a study showed that subanesthetic doses of S-ketamine counteracted the respiratory depression produced by opioids (Jonkman et al., 2018). This may result from S-ketamine antagonism of NMDA receptors, which enhances the sensitivity of the respiratory center to CO_2_ and excites sympathetic nerves, both of which may counteract respiratory depression (Jonkman et al., 2018). Furthermore, low doses of S-ketamine used for the induction of anesthesia preserve the patient’s pharyngeal and laryngeal reflexes (Sheikh and Hendry, 2018), which may greatly reduce the occurrence of respiratory depression caused by posterior tongue drop and increased airway secretions. High-dose use of opioids can produce hyperalgesia. The mechanism of this hyperalgesia is complicated and may be mediated by the activation of the central glutamine system caused by the activation of the μ receptor and the activation of the descending pain promotion mechanism (Lee et al., 2011; Colvin et al., 2019). The antagonistic effect of S-ketamine on NMDA receptors reduces the activation of the central glutaminergic system (Yu et al., 2016) and activates the descending analgesic pathway (Bosma et al., 2018), thus inhibiting the onset of this nociceptive hyperalgesia. Therefore, clinical reports of nociceptive hypersensitivity induced by S-ketamine are rare, and it has been found in studies that low-dose S-ketamine infusions attenuate the nociceptive hypersensitivity produced by opioids (Koppert et al., 2003; Bornemann-Cimenti et al., 2016). Although κ receptors are not involved in the analgesic process of S-ketamine, they seem to be associated with the psychiatric side effects produced by these processes. The reason is that S-ketamine produces dissociative and hallucinogenic effects similar to those of κ-receptor agonists, and such psychiatric symptoms can be partially blocked by κ-receptor antagonists (Nemeth et al., 2010; MacLean et al., 2013).

## 5 Monoamine system

### 5.1 Sympathetic excitation and analgesia

The sympathetic excitatory effects of S-ketamine are related to two aspects: 1) S-ketamine blocks sodium channels in parasympathetic neurons in the brainstem and inhibits parasympathetic activity; 2) S-ketamine inhibits NO release and enhances sympathetic activity (Okamoto et al., 1994; Irnaten et al., 2002). Sympathetic activation can increase noradrenaline (NA) and catecholamine levels in the body, leading to an increase in heart rate and blood pressure, increased cardiac output, and diastole of the bronchi (Takki et al., 1972; Robinson, 1985; Kamp et al., 2021). In addition to circulatory effects, NA binding to adrenergic receptors induces the release of endogenous opioids and produces analgesic effects (Romero et al., 2018), which is perhaps a pathway for the ketamine-induced increase in endogenous opioids (Pacheco Dda et al., 2014; Petrocchi et al., 2019). S-ketamine likely also exerts analgesic effects through the descending analgesic pathway (Bosma et al., 2018; Rogachov et al., 2019). The important transmitters of the descending analgesic pathway include 5-hydroxytryptamine and NA, both of which can promote release by ketamine, providing further evidence supporting this possibility (Takki et al., 1972; Pham and Gardier, 2019).

### 5.2 Other effects of the monoamine system

The psychiatric side effects produced by S-ketamine may be closely related to the increased release of central dopamine. Using positron emission tomography, Hashimoto et al. observed an increase in striatal dopamine release in monkeys after a single infusion of 0.5 mg/kg S-ketamine, which they suggested could be one of the reasons for the induction of acute psychotropic phenomena by S-ketamine (Hashimoto et al., 2017). However, studies also showed that R-ketamine did not cause an increase in striatal dopamine release, and no clinical manifestations of this psychotropic-like effect were observed with R-ketamine alone in clinical trials (Vollenweider et al., 1997; Hashimoto et al., 2017). Dopamine increases in the nucleus ambiguus usually promote synaptic plasticity in the limbic system of the midbrain and lead to addiction, but this is not the case for ketamine (Simmler et al., 2022). Although the deinhibition of dopaminergic neurons in the ventral tegmental area of mice by ketamine causes transient dopamine increases in the nucleus ambiguus, this deinhibition can be quickly terminated by the type-2 dopamine receptors on dopaminergic neurons, preventing the onset of synaptic plasticity and therefore leading to less susceptibility to ketamine addiction (Simmler et al., 2022). Dopamine does not appear to be involved in ketamine-induced vomiting, as studies have found that the type 2 dopamine receptor (D2) antagonist metoclopramide does not reduce the occurrence of ketamine-induced vomiting (Lee et al., 2012). However, administration of 5-hydroxytryptamine receptor antagonists reduced the incidence of vomiting after ketamine administration from 12.6% to 4.7%, suggesting that 5-hydroxytryptamine may be involved in its development in nausea and vomiting (Langston et al., 2008).

## 6 Cholinergic system

Cholinergic neurons control the release of acetylcholine by acting on nicotinic and muscarinic receptors and play an important role in a variety of human functions, such as arousal, cognition, learning, and memory (Leung and Luo, 2021). Ketamine can participate in the anesthetic process by modulating brain acetylcholine levels and affects patients’ postoperative cognitive level (Leung and Luo, 2021). In general, higher concentrations of acetylcholine are associated with cortical activation, such as during wakefulness and rapid eye movement (Marrosu et al., 1995). Sensitivity to ketamine-induced loss of consciousness can be increased when the acetylcholine transporter protein genetic gene is knocked out in mice and the concentration of cortical acetylcholine is reduced (Leung et al., 2021). Unlike the effects of anesthetics acting on GABA receptors, acetylcholine concentrations in the cerebral cortex in rats are significantly elevated after ketamine-induced loss of consciousness (Pal et al., 2015; Pal and Mashour, 2021). Moreover, the elevated acetylcholine concentration in the cerebral cortex may be one of the reasons for the susceptibility to excessive dreaming as well as hallucinations with ketamine (Pal and Mashour, 2021). While elevating acetylcholine concentrations, ketamine also inhibits muscarinic acetylcholine receptor function, mediating effects such as sympathetic excitation and bronchodilation (Gateau et al., 1989; Durieux, 1995). S-ketamine has a twofold higher affinity for muscarinic acetylcholine receptors than R-ketamine; however, *ex vivo* studies revealed that the relaxing effect of R-ketamine on airway smooth muscle is stronger than that of S-ketamine, which may be related to the different calcium channels affected by the two isomers (Pabelick et al., 1997; Zanos et al., 2018). In addition, antagonism of central acetylcholine by ketamine may mediate some ocular effects, such as nystagmus and blurred vision, which are mainly produced by S-ketamine and can be inhibited by a cholinesterase inhibitor derived from toxic lentils (Toro-Matos et al., 1980; Zanos et al., 2018).

## 7 Ion channel

### 7.1 HCN1

Hyperpolarization-activated cyclic-nucleotide-gated potassium channel 1 (HCN1) is closely associated with the hypnotic and amnesic effects of anesthetics (Zhou et al., 2015). HCN1 also plays an important role in the anesthetic process of S-ketamine and may be a molecular substrate for the hypnotic effect (Chen et al., 2009). The inhibition of HCN1, combined with the inhibition of NMDA receptor channels, produces a switch in the active population of excitatory neurons, which leads to a substantial reorganization of cortical activity (inhibition of active neurons and activation of silent neurons), including somatosensory areas and other primary senses (touch and vision), resulting in a “dissociative anesthetic” effect in which the brain is detached from its environment (Zhou et al., 2013; Cichon et al., 2023). In addition, HCN1 plays an important role in the regulation of cardiac pacemaker activity and rhythmic neuronal activity, and inhibition of HCN1 may induce bradycardia and cardiac arrest (Lee and MacKinnon, 2017; Shimizu et al., 2022). However, the inhibitory effect of S-ketamine on HCN1 in clinical use can only be observed as transient cardiac depression after administration, as it is quickly masked by the increase in heart rate produced by the activation of sympathetic excitation (Kamp et al., 2021). Recently, it has also been found that HCN1 is closely related to analgesia and can be used as a target for the production of novel analgesics (Ramírez et al., 2018), and whether S-ketamine can exert analgesic effects through this pathway remains to be investigated.

### 7.2 Na^+^ channels

S-ketamine also blocks Na^+^ channels. The blockage of Na^+^ channels in parasympathetic neurons in the brainstem may inhibit parasympathetic nerves and increase heart rate and blood pressure (Irnaten et al., 2002). Similarly, blockade of Na^+^ channels by S-ketamine can also produce inhibitory effects on sensation and movement and is used clinically as an adjuvant to local anesthetics (Frenkel and Urban, 1992; Nestor et al., 2022). However, due to the hydrophobic nature of ketamine, even though ketamine blocks Na^+^ channels in the central nervous system, this blockade does not achieve general anesthesia in the range of drug concentrations used clinically (Frenkel and Urban, 1992).

## 8 Current status of clinical application

S-ketamine has potent antidepressant effects and rapidly reduces suicidal ideation; it has been approved by the Food and Drug Administration (FDA) for use in adults with major depressive disorder and suicidal ideation and behavior (McIntyre et al., 2021). In the perioperative period, it can reduce the incidence of postoperative depression after orthopedic, gynecological, and gastrointestinal surgery (Wang et al., 2020; Min et al., 2023; Zhang et al., 2023) and is also effective in the control of *postpartum* depression (Han et al., 2022). S-ketamine also provides a potent analgesic effect, decreases postoperative Visual Analog Scale scores for a variety of surgical procedures, reduces opioid consumption, improves the quality of perioperative recovery (Argiriadou et al., 2004; Kadic et al., 2016; Miziara et al., 2016; Nielsen et al., 2019; Wang et al., 2020; Brinck et al., 2021; Han et al., 2022; Shen et al., 2022; Yu et al., 2022; Yuan et al., 2022; Zhu et al., 2022; Min et al., 2023), deters the onset of chronic pain and reduces postoperative opioid dependence (Nielsen et al., 2019). The incidence of hypotension and bradycardia is lower when S-ketamine is combined with propofol for sedation and analgesia (Huang et al., 2023), enhancing sedation and reducing both the dose of propofol and propofol-induced injection pain (Eberl et al., 2020; Xu et al., 2022). S-ketamine can also shorten the recovery time from anesthesia for painless examinations or operations, with lower respiratory and circulatory-related risks (Yang et al., 2022; Xin et al., 2022; Yongping et al., 2022; Zheng et al., 2022). It is equally safe and effective in pediatric patients (Zhang et al., 2022), and S-ketamine may also reduce the incidence of postoperative agitation as well as delirium in children (Li et al., 2022; Zhang et al., 2022; Chen et al., 2023).

S-ketamine is widely used in clinical practice, and its unique advantages in intraoperative maintenance and postoperative analgesia are gradually being explored. It has also been gradually accepted by anesthesiologists in pediatric-assisted sedation surgery or examination and has developed a variety of usages, such as nasal and sacral administration (Marhofer et al., 2000; Martindale et al., 2004; Lu et al., 2021). But as we explore the value of the drug, researchers must remain vigilant to the potential for abuse liability and long-term adverse events, for which there are insufficient data.

## 9 Conclusion

The mechanism of action of S-ketamine is quite complex. Antagonism of NMDA receptors is the basis for the various pharmacological properties of S-ketamine, such as dissociative anesthesia and analgesia, and S-ketamine also interacts with other receptors or channels to exert various pharmacological properties. NMDA receptors contain two subunits, GluN2A and GluN2B. S-ketamine acts on the GluN2A subunit primarily to induce hypnosis and on the GluN2B subunit primarily as part of the analgesic process. To produce analgesic effects, S-ketamine also activates opioid receptors. S-ketamine also excites sympathetic nerves and causes the release of NA by blocking sodium channels, inhibiting NO release and inhibiting muscarinic acetylcholine receptors, which in turn promotes the release of endogenous opioids and activates opioid receptors to produce further analgesic effects after binding to receptors. In addition, an increase in NA and 5-hydroxytryptamine also enhances the analgesic effect by activating the descending analgesic pathway. Inhibition of Ca^2+^ hyperpermeability reduces thalamic-cortical signal communication, which can produce loss of consciousness and analgesic effects. However, this inhibition also leads to Ca^2+^ dysregulation, activation of glutamate nondependent pathways, induction of Ca^2+^ inward flow and mobilization of calcium pools, elevation of Ca^2+^ concentration in the neuronal cytoplasm of the brain, and consequently to dopamine release, which induces euphoria and psychotropic side effects. Antagonism of NMDA receptors may also indirectly cause sustained activation of AMPA receptors, which enhances thalamocortical and cortical synaptic transmission leading to distinct EEG features and may contribute to dissociative anesthesia. As a potential analgesic target and hypnotic substrate for S-ketamine, HCN1 may also participate in the process of dissociative anesthesia in concert with NMDA receptors. Most of the current related studies are carried out using racemic ketamine and the effect of R-ketamine in racemic ketamine has not been fully elucidated; regardless of its clinical analgesic and anesthetic effects or related studies on the affinity of various receptors, the analgesic and anesthetic effects of racemic mixed ketamine are mainly mediated by S-ketamine. Therefore, in the absence of direct evidence of S-ketamine, racemic ketamine’s mechanism in analgesia and anesthesia can be partially explained by the mechanism of S-ketamine. In summary, S-ketamine has been shown to have stronger analgesic effects and fewer side effects than ketamine and has better clinical application prospects as an anesthetic drug; the mechanisms of action of S-ketamine deserve in-depth study.
